# Integrating horticultural practices with smallholder resources improves Rosa roxburghii yield and nitrogen use efficiency

**DOI:** 10.1038/s41598-025-15403-z

**Published:** 2025-08-11

**Authors:** Pengqiang Wang, Fan Shi, Pengbo Dong, Yiwei Jian, Zhilong He, Xiaoqiang Jiao

**Affiliations:** 1 Sanya Institute of China Agricultural University, Sanya, 572000 China; 2 State Key Laboratory of Nutrient Use and Management, College of Resources and Environmental Sciences, Key Laboratory of Plant-Soil Interactions of MOE, China Agricultural University, Beijing, 100193 China

**Keywords:** Environmental sciences, Environmental social sciences

## Abstract

*Rosa roxburghii* Tratt is one of the main cash crops in mountainous areas of Guizhou where production is a large contributor to the income of smallholders. In Guizhou Province, cultivation of R. *roxburghii* is largely dominated by smallholders employing extensive management practices, which typically results in low yield and economic challenges. Moreover, the effect of integrated and localized horticultural practices and smallholder resource endowment on yield and resource use efficiency is not well understood. In this study, localized technology and nitrogen use efficiency were assessed for 121 smallholders growing *R. roxburghii* in the main production zone in Guizhou Province. The average yield of *R. roxburghii* among smallholders was 5.9 t ha^-1^. Based on yield and nutrient use efficiency, smallholders were categorized into four groups. The yield of the high-yielding and high-efficiency (HH) group was 8.4%, 99.1%, and 97.8% higher than that of the high-yielding and low-efficiency (HL), low-yielding and high-efficiency (LH), and low-yielding and low-efficiency (LL) groups, respectively. The partial factor productivity of the HH group’s Nitrogen fertilizer (PFP-N) was 501.6%, 31.7%, and 589.0% higher than that of the HL, LH, and LL groups, respectively. Educated smallholders in the HH group employed optimized horticultural practices, including chemical fertilizer, pruning, pest control, planting density, and weed control. Thus, smallholder farm-led *R. roxburghii* crop production demonstrates great potential for high yield and efficient nitrogen use through the coupling of localized technology and the resource endowment of smallholders.

**Supplementary Information:**

The online version contains supplementary material available at 10.1038/s41598-025-15403-z.

## Introduction

*Rosa roxburghii*, commonly known as *song chungui* and *mu lizi*, is a perennial deciduous fruit tree belonging to the family Rosaceae^1^. These highly valued crops are also known as the “king of vitamin C” and are a source of income for smallholder farms^2,3^. *R. roxburghii* crops are widely distributed in Guizhou, Sichuan, and other regions in China; however, Guizhou Province has the largest distribution and output in China^4^. According to a recent survey, the core production area of *R. roxburghii* in Guizhou reached 1.17 × 10^5^ ha in 2020, accounting for 92.6% of the country’s production^5^. At present, *R. roxburghii* cultivation in most areas of Guizhou Province remains dominated by smallholders, who generally lack access to advanced education, information, and technological resources. Moreover, the production of *R. roxburghii* faces problems of limited yields and suboptimal resource efficiency^6^. Optimizing resource utilization is critical to achieving higher yields and ensuring sustainable intensification of production, which in turn improves smallholders incomes. Therefore, efforts to standardize and improve cultivation practices for R. *roxburghii* are urgently needed^7^.

Several studies have evaluated existing horticultural practices to improve the sustainability of *R. roxburghii* crop production. Fan et al. (2001) explored the method of whip-back pruning, which could significantly promote the growth of *R. roxburghii* and improve its yield and quality. Reasonable shaping and pruning to adapt the tree size and canopy structure to the planting density can not only improve canopy ventilation, light transmission, and yield but also reduce ineffective transpiration and water consumption, thereby improving water use efficiency^8,9^. According to Han et al. (2021), powdery mildew is a major disease that significantly hinders the growth of R. *roxburghii*. Effective control through integrated pest management strategies is necessary to improve the output and quality of *R. roxburghii* crops^10^. The effects of different pH conditions on the absorption characteristics of different nitrogen forms in *R. roxburghii* have also been studied to guide for rational fertilizer use^11^. *R. roxburghii* intercropped with corn and pepper has been found to promote soil macro-aggregate formation, thereby enhancing crop yield and quality^12^. In addition to studies that focused on management and protection (such as fertilization, weeding, and pest control), standardized cultivation practices have been gradually refined to offer technical support for the sustainable production of R. *roxburghii*. However, such research is based on experimental plot management rather than farmer-led field trials, and these practices are difficult to implement by smallholders.

Implementing localized technologies is a complex process that involves multiple dimensions, including the experience of smallholders and the cultivation, management, and care of *R. roxburghii* crop and the agriculture market. According to Ma (2021), smallholders’willingness to grow grain is influenced by factors such as age and education level. However, that study focused solely on motivational aspects and overlooked the role of management technologies^13^. Cai and Tang (2013) showed that the adoption of agricultural technology depends on the resource endowments of smallholders^14^. To date, no studies have systematically examined the integration of smallholder resource endowments with management technologies. While the effective use of technology by smallholders is key to improving the sustainable production of *R. roxburghii*, the structure and function of localized production systems led by smallholders remain poorly understood.

Herein, 121 smallholders were surveyed in Qiannan, Guizhou Province, and the cultivation, management, and maintenance practices of *R. roxburghii* were assessed. The objectives were to identify the main limiting factors affecting the synergistic realization of high yield and efficiency in R. *roxburghii* production by smallholders and to explore potential horticultural practices to achieve synergistic improvements in both yield and efficiency.

## Materials and methods

### Overview of the research area

The study was conducted in Cha-xiang village, Longli County, Guizhou Province (26°56’N, 107°37’E). Cha-xiang Village is located north of Gujiao Town, along Qianxi road, 17 km from the town government and 31 km from Guiyang City. The forest coverage rate of Cha-xiang Village is exceeding 80% and is recognized as an important water conservation area in China; however, it is also a village with severe water shortages, less paddy fields and more dry land. The region has an annual average temperature of 14.8° C and an annual average rainfall of 1100 mm. Cha-xiang Village is also called the “ten-mile *Rosa roxburghii* ditch” because of its approximately 1000 hectares of *R. roxburghii* crops^15^. It serves as a major production base for R. *roxburghii*, holding the largest planting area and output in China. The crop has become the cornerstone of the village’s economy, playing a critical role in lifting local smallholders out of poverty and promoting financial stability and well-being.

### Survey of smallholders

Our survey included the following four aspects: (1) Sample selection: 138 growers were randomly selected for questionnaire surveys among more than 557 *R. roxburghii* growers in Cha-xiang Village, Longli County. In total, 121 valid questionnaires were obtained, representing a planting area of 141.8 ha (with an average of 7.3 fragmented plots per household), which accounted for approximately 4.2% of the local planting area. All R. *roxburghii* in the area were of the Guinong No.5 variety, and planting management was extensive, without irrigation. (2) Questionnaire design: this primarily included gathering basic farmer information (such as gender, age, and educational level), crop details (planting time and orchard tree density), and orchard management practices (fertilizer application, fertilization period, fertilization type, frequency of orchard clip, weed control, and other related information). The fertilizer used by smallholders in this study was potassium sulfate compound fertilizer (15-15-15), distributed by the government. (3) Implementation of the survey: the survey was conducted from November to December 2021; surveyors completed the questionnaire through face-to-face interviews. (4) Data revision: owing to the limited knowledge of smallholders, their understanding of fruit tree management, and the degree of cooperation in research work, the data and information obtained in the survey could potentially differ from the actual situation. A question-and-answer approach was utilized to obtain reliable information on yield and details of horticultural practices. After conducting the survey, the data were revised and, if necessary, a return survey was conducted by telephone (The raw survey data are available in Supplementary Dataset S1 (Survey Data.xlsx).

All methods were performed in accordance with the relevant guidelines and regulations.

Ethics approval and consent to participate: The survey protocol was approved by the Ethics Committee of China Agricultural University. All participants provided verbal informed consent.

### Data calculation

#### Partial factor productivity of nitrogen fertilizer

Partial factor Productivity of Nitrogen fertilizer (PFP-N), which is one of the indicators for the classification of smallholders along with yield, was calculated as follows:

$$\:\text{P}\text{F}\text{P}-\text{N}=\text{Y}/\text{F}\text{N}$$

where $\:\text{P}\text{F}\text{P}-\text{N}$ is the partial productivity of N fertilizer (kg kg^−1^), Y is the yield of *R. roxburghii* (t hm^−2^), and FN is the N input (t hm^−2^). PFP-N refers to the crop yield ratio to nitrogen application rate^16^. Although some nitrogen was lost during nitrogen fertilizer application, smallholders ignored the nitrogen fertilizer application efficiency and only cared about the relationship between nitrogen application rate and *R. roxburghii* yield. Therefore, the concept of partial nitrogen fertilizer productivity was used in this study^17^.

The nutrient content of chemical fertilizers was determined based on either the standard nutrient composition or the nutrient content marked on the fertilizer bag. Meanwhile, manure nutrients were converted using the reference values provided in “A Guide to Fertilization of Major Crops in China” and all values were expressed in terms of equivalent amounts of pure nutrients^18^.

#### Data processing and statistical analysis

Data were collated, calculated, and plotted using Microsoft Excel 2010 (Microsoft Corporation, Redmond, WA, USA). Drawings were prepared using Origin 2021 software (Origin Lab, Northampton, MA, USA).

Spss software (IBM SPSS, Chicago, IL, USA) was used to conduct ANOVA on the data of (a) and (b) in Fig. 2 and (a) in Fig. 3, and LSD method was used to compare whether there were differences between different treatments, and the corresponding letters were marked in the figure. Structural equation modeling (SEM) performed with SPSS Amos 26.0 software (IBM SPSS, Chicago, IL, USA) was used to evaluate the causal relationship between smallholder resource endowment and horticultural management practices and yield. The adequacy of the models was tested using the maximum likelihood-ratio chi-square (*p* > 0.05) goodness-of-fit test, the comparative fit index (CFI > 0.90), and the root mean square error of approximation index (RMSEA < 0.08)^19^.

## Results

### Classification of smallholders

**Fig. 1:**
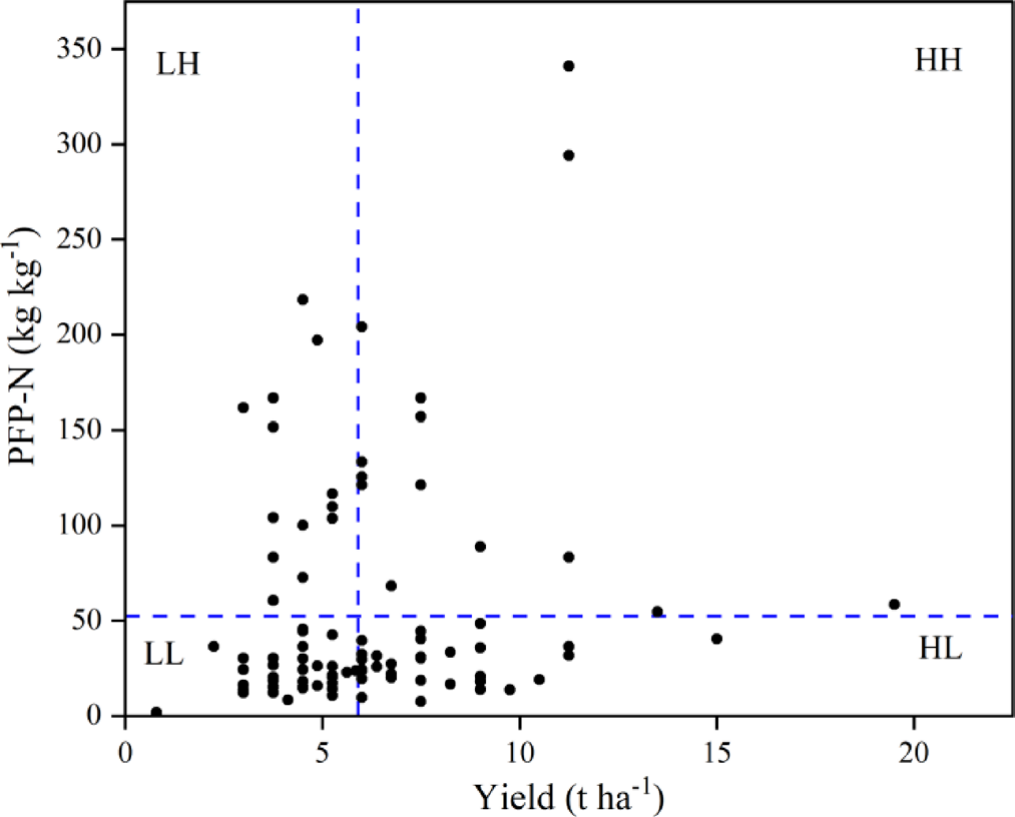
Relationship between yield (fresh weight) of *R. roxburghii* and partial factor of production (PFP-N) (yield per unit of N use) in Cha-xiang Village, Longli County, Guizhou Province. The blue dotted lines represent the average yield and PFP-N of *R. roxburghii* production by 121 smallholders surveyed in this study. Smallholders were divided into four groups based on yield and PFP-N as follows^20^: LL, low-yielding and low-efficiency, LH: low-yielding and high-efficiency, high-yielding and low-efficiency (HL), and high-yielding and high-efficiency (HH).

**Fig. 2:**
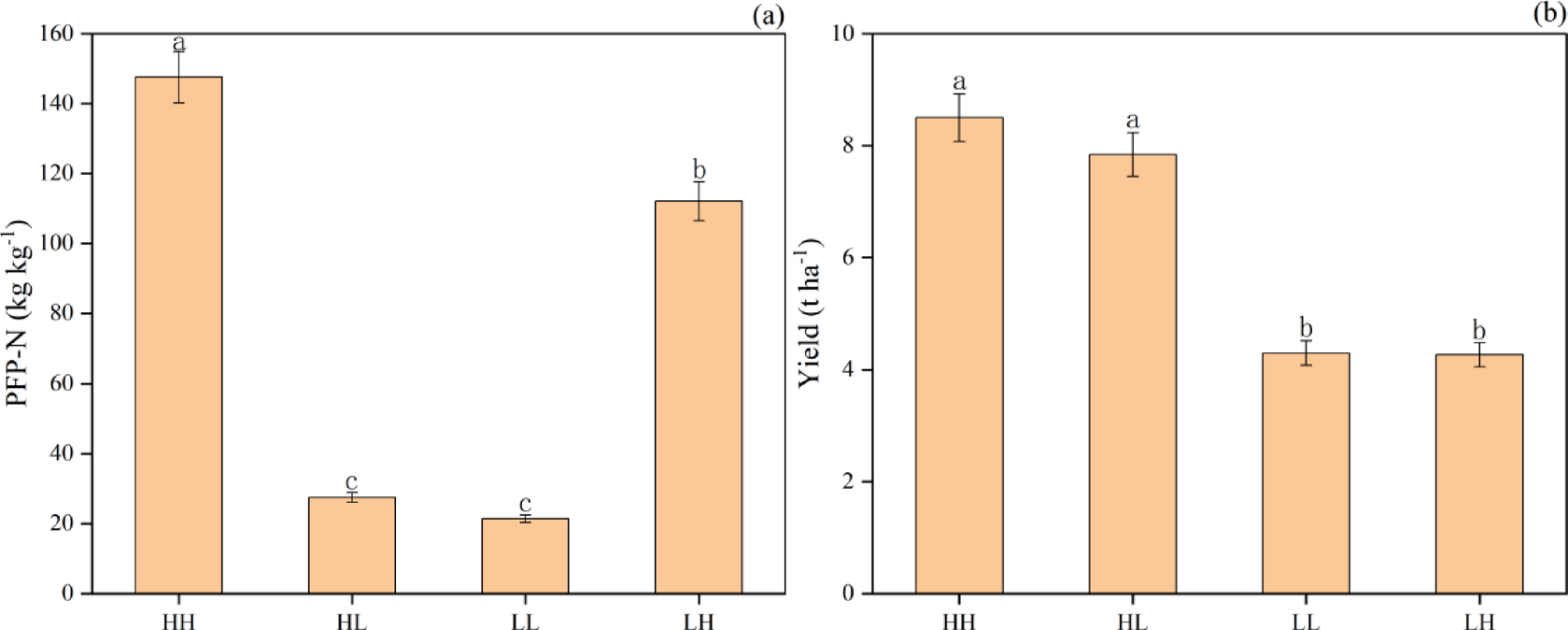
Average PFP-N **(a)** and yield **(b)** of *R. roxburghii* among the different groups of smallholders. Error bars indicate standard error (SE). Different letters represent statistically significant differences based on LSD (Least Significant Difference) test at *P* < 0.05. LL, low-yielding and low-efficiency, LH: low-yielding AND high-efficiency, high-yielding and low-efficiency (HL), and high-yielding and high-efficiency (HH).

Based on observed yield and Partial factor Productivity of Nitrogen fertilizer (PFP-N) of *R. roxburghii* smallholders were classified into four groups using the quartile method, with the average yield and average PFP-N serving as thresholds. Of the 121 smallholders, 15, 37, 18, and 51 were classified as high-yielding and high-efficiency (HH) group, high-yielding and low-efficiency (HL) group, low-yielding and high-efficiency (LH) group and low-yielding, and high-efficiency (LL) group, respectively (Fig. 1). The yield of smallholders in the HH group was 8.4%, 99.1%, and 97.8% higher than that of the HL, LL, and LH groups, respectively. In addition, the nitrogen use efficiency in the HH group exceeded that of the HL, LH, and LL groups by 501.6%, 31.7%, and 589.0%, respectively (Fig. 2).

### Horticultural management practices of different smallholders’ groups

**Fig. 3:**
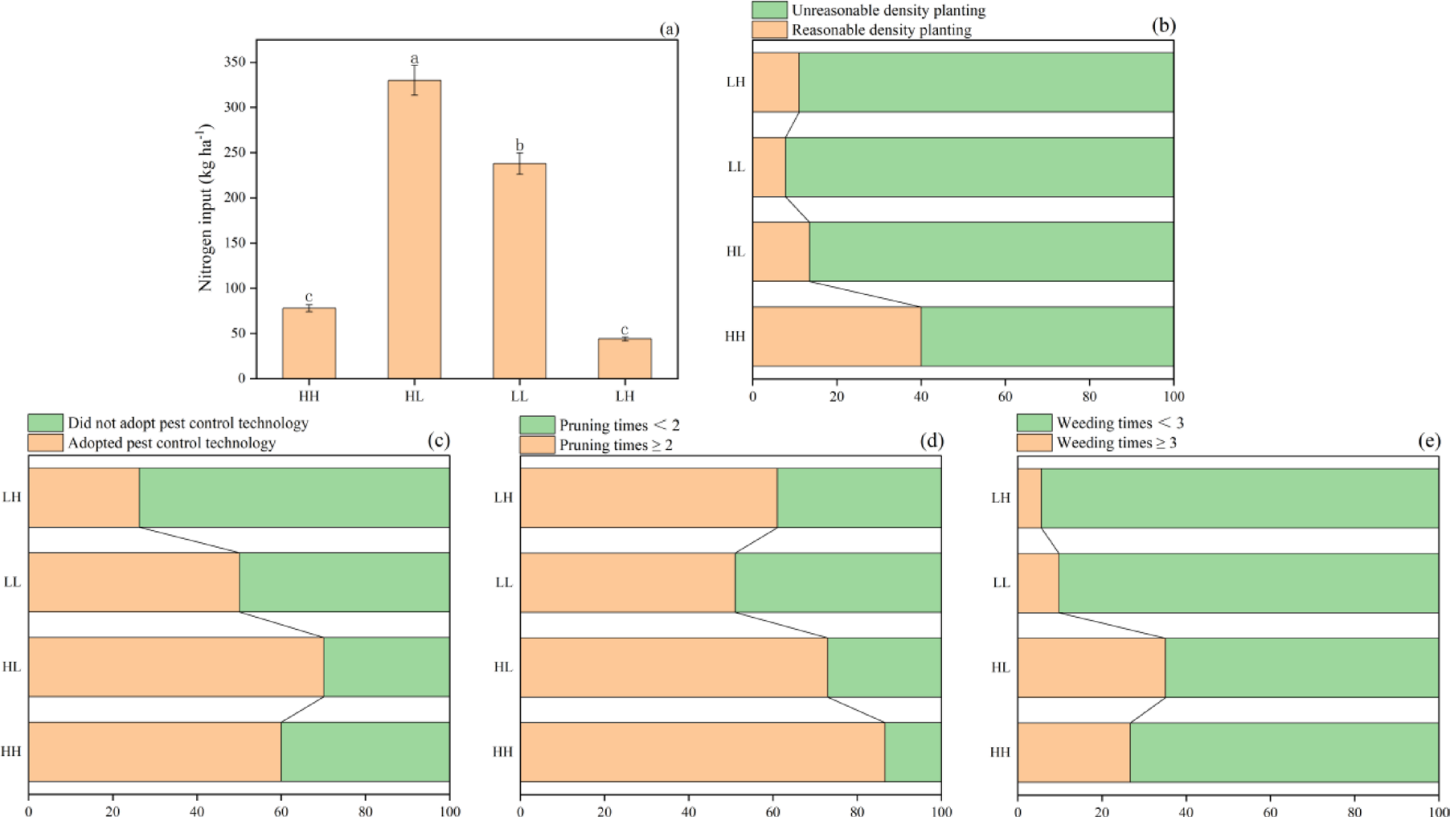
Relative chemical nitrogen use **(a)**, planting density **(b)**, pest control adoption rate **(c)**, and number of pruning times **(d)** and weed control times **(e)** of *R. roxburghii* production among the different smallholder groups. Error bars indicate standard error (SE). Different letters represent statistically significant differences based on LSD (Least Significant Difference) test at *P* < 0.05. LL, low-yielding and low-efficiency; LH: low-yielding and high-efficiency, high-yielding and low-efficiency (HL), and high-yielding and high-efficiency (HH).

There were differences in horticultural management practices among the different smallholder classifications. Regarding nitrogen inputs, on average, per hectare of orchard, the HH group used 252 kg and 160 kg less pure nitrogen than that of the HL group and LL groups, respectively, but 36 kg more than that of the LH group. Regarding the planting density of *R. roxburghii*, the proportion of orchards with reasonable tree density in the HH group was 26.5%, 29.0%, and 29.5% higher than that in the HL, LH, and LL groups, respectively. The proportion of smallholders adopting pest control technology in the HH group was 33.7% and 9.9% higher than that in the LH and LL smallholder groups, respectively, but 10.1% lower than the HL group. The proportion of smallholders in the HH group who pruned *R. roxburghii* at least twice times per year was 25.5%, 28.7%, and 35.6% higher than the HL, LH, and LL groups, respectively. With respect to weed control, the proportion of smallholders in the HH group who implemented weed control practices at least three times per year was 21.1% and 16.9% higher than the LH and LL groups but 8.4% lower than the HL group (Fig. 3).

### Resource endowment of different smallholder groups

**Fig. 4:**
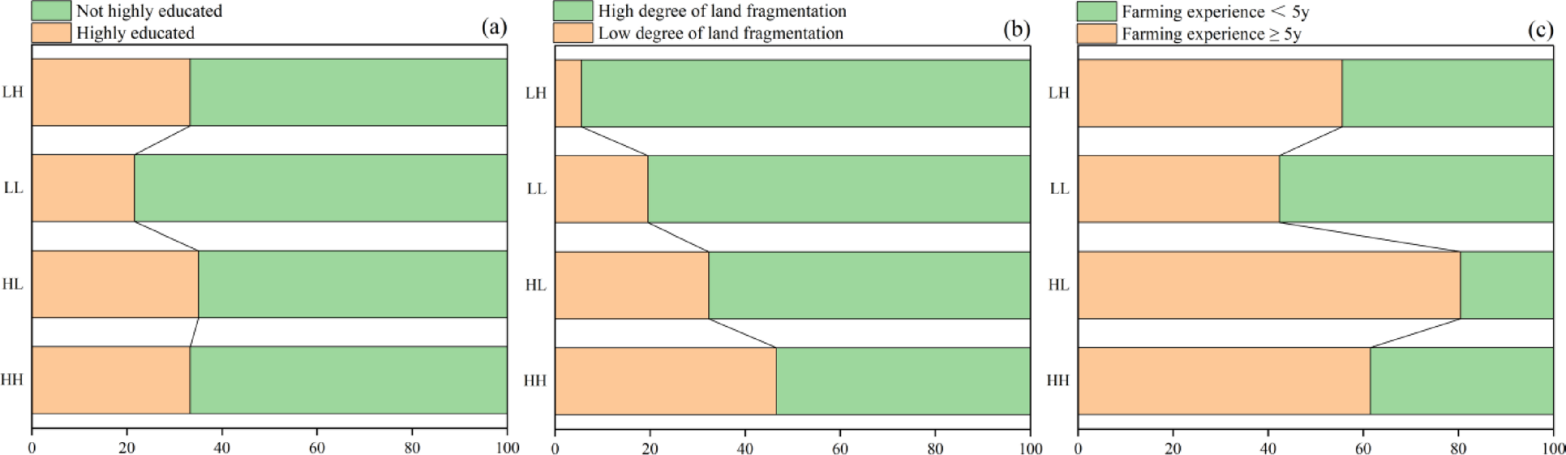
Relative levels of education **(a)**, land fragmentation **(b)**, and farming experience **(c)** among the different smallholder *R. roxburghii* production groups. LL, low-yielding and low-efficiency, LH: low-yielding and high-efficiency, high-yielding and low-efficiency (HL), and high-yielding and high-efficiency (HH).

Smallholder resource endowment severely affected the production performance of *R. roxburghii* in this study. Differences were observed among the four groups in terms of resource endowment (land fragmentation, farmer experience, and education level of smallholders). The HH group had the same percentage of highly educated people as the LH group; however, it was 1.7% higher than the LL group and 1.8% lower than the HL group. The proportion of land fragmentation in the HH group was 14.2%, 41.0%, and 27.0% lower than that in the HL, LH, and LL groups, respectively. Smallholders with more than five years of planting experience were considered to have rich cultivation experience. The proportion of smallholders in the HH group who grew *R. roxburghii* for over five years was 5.9%, which was 19.1% higher than that in the LH and LL groups but 19% lower than that in the HL group (Fig. 4).

### Effect of synthetic variables on yield

**Fig. 5:**
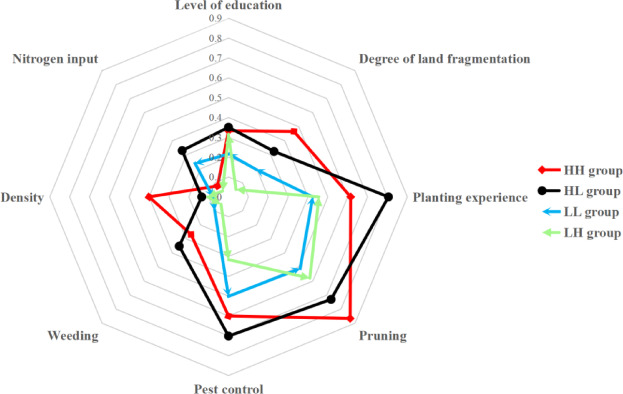
Factors influencing the production performance of *R. roxburghii* among different groups of smallholders in Cha-xiang Village, Longli County, Guizhou Province. LL, low-yielding and low-efficiency, LH: low-yielding and high-efficiency, high-yielding and low-efficiency (HL), and high-yielding and high-efficiency (HH).

All indicators are potential factors that ultimately affect *R. roxburghii* yield. According to the radar chart, the four groups of smallholders exhibited different patterns in both resource endowment and horticultural management practices (Fig. 5). Therefore, we identify the eight indicators (levels of education, degree of land fragmentation, planting experience, pruning, pest control, weeding, plant density, and nitrogen input) are the factors that ultimately influence yield and efficiency.

**Fig. 6:**
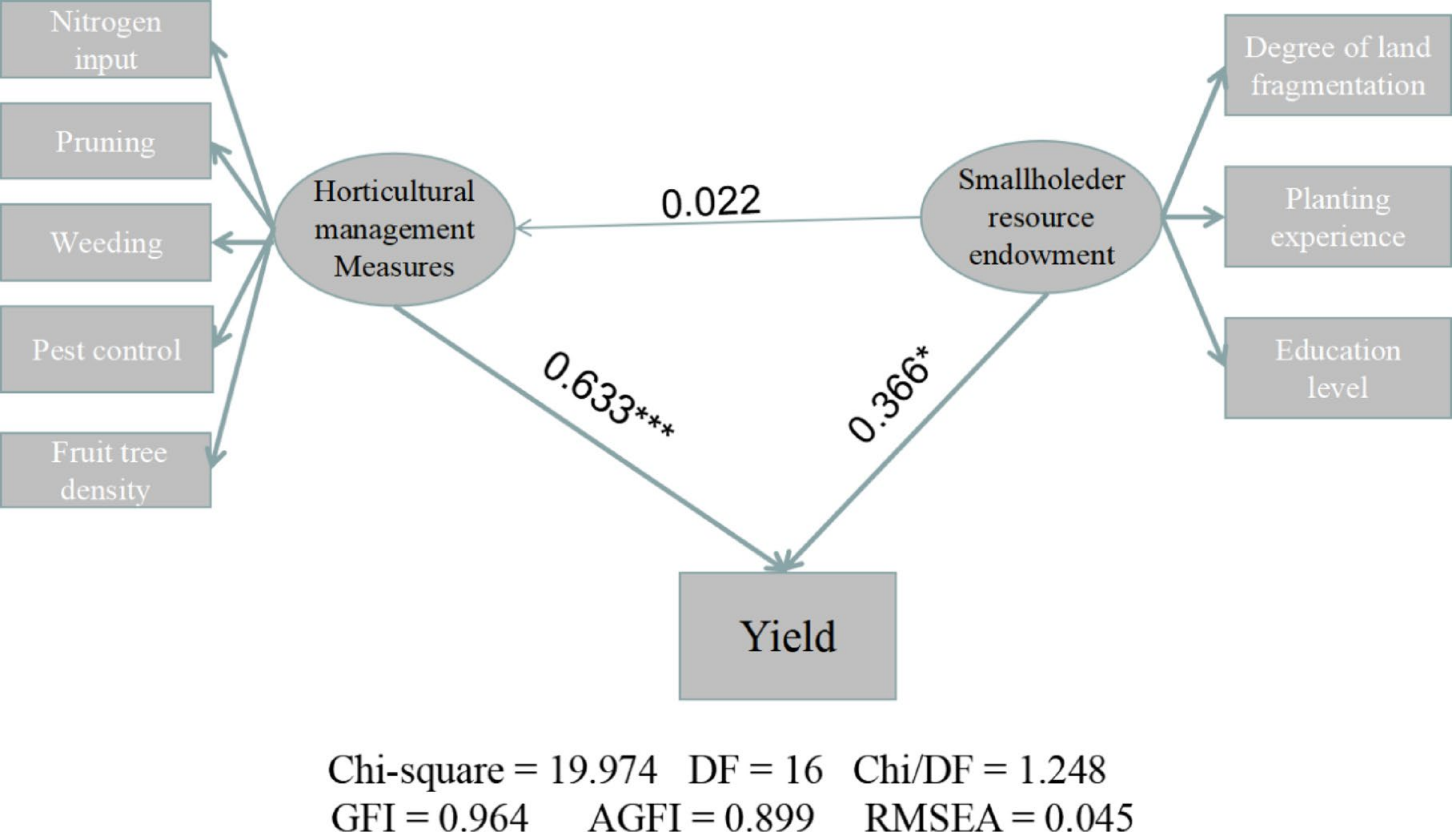
Structural equation model showing the relationship between resource endowment and horticultural management practices of smallholders and yield of *R. roxburghii*. *, *p* < 0.05; **, *p* < 0.01, and ***, *p* < 0.001.

The effect of horticultural management practices on yield was 0.633, and that of smallholder resource endowment was 0.366, both of which were significant (*p* < 0.05) (Fig. 6). In contrast, the effect of smallholder resource endowment on horticultural management practices was 0.022 and not statistically significant.

## Discussion

The dominance of smallholders in R. *roxburghii* cultivation has resulted in substantial variability in management practices. Despite differences in both management techniques and resource endowments among smallholders, 15 out of the 121 smallholders surveyed achieved both high yield and high nitrogen use efficiency, indicating the potential for broader replication of such outcomes under similar smallholder conditions. Analysis of the HH group revealed that these smallholders adopted more optimized management practices compared to other groups. On average, they applied 160 kg less N fertilizer per hectare than the LL group, and 252 kg less than the HL group. The proportion of smallholders with reasonable planting density in the HH group was 26.5%, 29.0%, and 29.5% higher than in the HL, LH, and LL groups, respectively. They also exhibited more proactive management practices, including pruning frequencies that were 25.5–35.6% higher and weed control activities that were 16.9–21.1% more frequent compared to the LH and LL groups. These quantitative differences suggest that the HH group employed more efficient and comprehensive horticultural practices for *R. roxburghii* cultivation. The characteristics of resource endowment—such as land fragmentation degree, planting experience and education level of smallholders in the HH group, are also superior to those of all surveyed smallholders. The combination of sound horticultural management practices and these favorable smallholder characteristics resulted in a high yield of *R. roxburghii* and high N use efficiency of smallholders in the HH group. Compared with HH, HL can synergistically achieve high yield and high N use efficiency of *R. roxburghii* by appropriately reducing fertilization, increasing planting density, and increasing pruning frequency. In this study, “optimal fertilization” is defined as a nitrogen input level that is relatively lower but achieves greater efficiency, as exemplified by the HH group. Specifically, HH smallholders applied 160 kg less nitrogen per hectare than those in the LL group, yet attained superior yield and nitrogen use efficiency. These findings suggest that excessive nitrogen application is unnecessary, and careful management of fertilizer inputs can significantly enhance production outcomes. In addition to optimal fertilization, achieving high yield and nitrogen use efficiency—particularly among smallholders in the LL group—also requires appropriate planting density, increased pruning frequency, more frequent weeding, and improved pest management.

Our study demonstrates that integrating localized horticultural practices with smallholder resource endowment is crucial for achieving synergistic improvements in R. *roxburghii* yield and nitrogen use efficiency. By analyzing yield and efficiency variations among smallholders with diverse management approaches and resource backgrounds, we found that this integration plays a significant role in explaining yield variability. Unlike prior research that focused on isolated techniques (e.g., pruning^8^; pest control^10^ or confined experimental trials (e.g., oxalic acid application^21^; water-fertilizer coupling^22^, our study identifies scalable, practical practices tailored to real-world smallholder contexts. This approach accounts for both the technology itself and the characteristics of smallholders, particularly the integration of the two; as such, it is both practical and readily implementable. Aligning horticultural practices with smallholder attributes facilitates technology adoption and promotes the sustainable production of *R. roxburghii*. Wang et al. highlighted that improving apple productivity and nutrient efficiency requires a clear understanding of yield potential, nutrient use, and optimal management^23^. Platforms like the Science and Technology Backyard can facilitate this knowledge transfer, enabling researchers to adapt techniques for local implementation^24^. Based on these findings, smallholders are able to effectively adopt localized horticultural practices tailored to their specific resource endowments, thereby achieving synergistic improvements in both yield and nitrogen use efficiency in R. *roxburghii* cultivation.

Although this study was conducted in Cha-xiang Village—the largest R. *roxburghii* production base in China with over 1,000 hectares under smallholder cultivation—its findings are likely generalizable to regions with comparable agroecological and socioeconomic conditions. The 121 randomly selected smallholders varied widely in education, land use, and management intensity, contributing to the robustness and representativeness of the dataset. Admittedly, time constraints and variability in data collection methods introduced some inconsistencies. Nonetheless, the results underscore the potential for smallholders to achieve both high yields and efficient nitrogen use through improved practices. Further studies in other major production areas, such as Sichuan and Yunnan, would help to validate and refine the broader applicability of these conclusions.

## Conclusion

A set of localized technical procedures was developed to enhance both yield and nitrogen use efficiency in *R. roxburghii* production. The observed differences in performance among smallholders were primarily attributed to variations in horticultural practices and resource endowments. Specifically, the most effective management practices included reduced nitrogen input, more frequent pruning and weeding, optimal planting density, and the adoption of pest control measures. In terms of resource characteristics, smallholders with less fragmented land, longer planting experience, and higher education levels tended to achieve better outcomes. These findings suggest that combining targeted horticultural strategies with favorable smallholder attributes holds great potential to improve *R. roxburghii* productivity and resource efficiency. This study highlights a practical and scalable approach to promoting sustainable *R. roxburghii* cultivation through the synergy of localized technology and farmer capacity in China.

## Supplementary Information

Below is the link to the electronic supplementary material.

Supplementary Material 1

## Data Availability

Data Availability: All data generated or analyzed during this study are included in this published article and its supplementary information files.

## Abbreviations

*R. roxburghii*: *Rosa roxburghii*
HH: high-yielding and high-efficiency
HL: high-yielding and low-efficiency
LH: low-yielding and high-efficiency
LL: low-yielding and low-efficiency
PFP-N: partial factor Productivity of Nitrogen fertilizer
SEM: structural equation modeling
CFI: comparative fix index
RMSEA: root mean square error of approximation index
